# Self-Cleaning Polyester Fabric Prepared with TiOF_2_ and Hexadecyltrimethoxysilane

**DOI:** 10.3390/polym13030387

**Published:** 2021-01-26

**Authors:** Euigyung Jeong, Heeju Woo, Yejin Moon, Dong Yun Lee, Minjung Jung, Young-Seak Lee, Jin-Seok Bae

**Affiliations:** 1 Department of Textile System Engineering, Kyungpook National University, Daegu 41566, Korea; wolfpack@knu.ac.kr (E.J.); whj4533@naver.com (H.W.); myjibsm@naver.com (Y.M.); 2 Department of Polymer Science and Engineering, Kyungpook National University, Daegu 41566, Korea; dongyunlee@knu.ac.kr; 3 Department of Chemical Engineering and Applied Chemistry, Chungnam National University, Daejeon 34134, Korea; hiddomi00@naver.com (M.J.); yslee@cnu.ac.kr (Y.-S.L.)

**Keywords:** TiOF_2_, self-cleaning fabrics, organic silane, photocatalyst, superhydrophobicity

## Abstract

In this study, self-cleaning polyester (PET) fabrics were prepared using TiOF_2_ and hexadecyltrimethoxysilane(HDS) treatment. TiOF_2_ was synthesized via direct fluorination of a precursor TiO_2_ at various reaction temperatures. The prepared PET fabrics had superior photocatalytic self-cleaning properties compared with anatase TiO_2_/HDS-treated PET fabrics under UV and sunlight with 98% decomposition of methylene blue. TiOF_2_/HDS-treated PET fabrics also had superior superhydrophobic self-cleaning properties compared with anatase TiO_2_/HDS-treated PET fabrics with a 161° water contact angle and 6° roll-off angle. After the self-cleaning tests of the non-dyed TiOF_2_/HDS-treated PET fabrics, we prepared dyed TiOF_2_/HDS-treated PET fabrics to test practical aspects of the treatment method. These PET fabrics were barely stained by tomato ketchup; even when stained, they could be self-cleaned within 4 h. These results suggest that practical self-cleaning PET fabrics with superhydrophobicity and photocatalytic degradation could be prepared using TiOF_2_/HDS-treatment.

## 1. Introduction

The development of innovative functional textile materials has attracted much research interest, and the fabrication of fabrics with a self-cleaning surface has been vigorously investigated [1,2,3]. Self-cleaning fabrics are eco-friendly because they can reduce the water consumption required for laundering clothes; they also have stain resistant and antimicrobial properties [4].

The development of self-cleaning fabrics has two components. The first is the fabrication of a superhydrophobic surface based on the famous lotus effect [5]. Superhydrophobic surfaces are usually observed in nature, such as in lotus leaves. Such surfaces are extremely water repellent with water contact angles of greater than 150°. Dirt on a superhydrophobic surface is washed off when the spherical water droplets roll off the surface [6,7,8]. The superhydrophobicity of a surface depends on surface roughness and energy. Therefore, many researchers have used low surface energy materials and/or increased surface roughness using various treatment methods [6,7,8,9,10,11,12,13]. The low surface energy materials applicable to this purpose are alkyl amines, silicates, organic silanes, and fluorinated silanes [6,7,8,9]. The treatment methods used for this purpose are electrospinning, plasma treatment, chemical vapor deposition, layer-by-layer deposition, polymerization reactions, colloidal template techniques, wet chemical reactions, self-assembly, and sol-gel [10,11,12,13,14,15,16,17,18,19,20,21]. The second component is fabrication of the photodegradable surfaces using photocatalysts, such as anatase TiO_2_ [22]. The photodegradable surface removes dirt via the light induced degradation of stained materials, which is expedited by the photocatalysts [22]. The most popular photocatalyst is anatase TiO_2_. It is sensitized by UV (ultraviolet) light and produces radicals via reaction with oxygen or water in air [4]. However, UV light is only a small component of sunlight and it is not efficient enough to be used in the real world. Therefore, much research in this field has focused on the development of visible light sensitized photocatalysts [23,24,25,26,27,28,29,30] since visible light is the largest component of sunlight. Such research included nonmetal elements, such as B, C, N, and S, doping of TiO_2_ and metals, such as Fe, Ag, doping of TiO_2_ [23,24,25,26]. Dye and TiO_2_ composites are also used in this approach [27,28,29,30].

Recently, self-cleaning fabrics with superhydrophobicity and photocatalytic degradation have been reported [3,4,31,32,33,34,35]. These were prepared via the treatment of photocatalysts followed by the treatment of organic silanes. This approach produced excellent stain resistant surfaces owing to their superhydrophobicity and photocatalytic removal of stains due to the surface photocatalyst being sensitized by visible light. However, their application in the textile industry seems limited because the functional properties of fabrics are given by the textile finishing process, which uses fabrics dyed with a particular color. When the photocatalysts are sensitized by visible light, they absorb visible light, resulting in their own color. It has not yet been confirmed whether the color of the photocatalyst affects the color of the dyed fabric.

Therefore, the present study aimed to investigate the practical aspects of the fabrication of self-cleaning fabrics using TiOF_2_ and hexadecyltrimethoxysilane (HDS) treatment. TiOF_2_ is a visible light sensitizable photocatalyst with greater photocatalytic capability than TiO_2_ [36]. TiOF_2_ was synthesized via direct fluorination of a precursor TiO_2_ at various reaction temperatures. The prepared TiOF_2_ was applied to standard white polyester (PET) fabric, which is one of the major textile fabrics used, and then HDS was applied onto the prepared photocatalyst-treated PET fabrics. The superhydrophobicity and photocatalytic degradation of the prepared fabrics were investigated. For the first time, based on the best results obtained for the prepared self-cleaning white PET fabrics, a dyed PET fabric was treated with the photocatalyst and HDS, and its practical self-cleaning properties toward tomato ketchup were tested.

## 2. Materials and Methods

### 2.1. Materials

PET standard fabric was purchased from Test Fabrics Inc. TiO_2_ powder (Titanium(IV) oxide, 98+%, anatase powder) was purchased from Acros Organics (Geel, Begium). Titanium(IV) isopropoxide (97%, TTIP) and hexadecyltrimethoxysilane (≥85%) were purchased from Sigma-Aldrich (St. Louis, MO, USA). Isopropanol (99%, IPA) was purchased from Samchun (Seoul, Korea) and methylene blue (MB) hydrate (>70%) was purchased from Tokyo Chemical Industry (Tokyo, Japan).

### 2.2. Synthesis of TiOF_2_ Photocatalysts

First, 1 mole of TTIP, 100 mL of IPA, and 4 moles of distilled water were stirred for 30 min and then, the temperature of the mixture was raised up to 50 °C and stirred for 12 h to produce the TiO_2_ precursor. The TiO_2_ precursor was mixed with IPA and dried in the oven at 60 °C for 8 h to remove impurities. The dried precursor was placed in a nickel boat and then into a lab-made direct fluorination reactor, which has been reported in the previous research [36]. Next, the temperature of the reactor was raised to 300, 350, 400, 450, 500, or 550 °C and maintained for 30 min with 0.2 bar of F_2_ gas (99.8%, Messer Griesheim Gmbh, Bad Soden, German) and 0.8 bar of N_2_ gas (99.999%, Messer Griesheim Gmbh, Bad Soden, German) for fluorination. The reactor was cooled and purged three times with N_2_ gas.

### 2.3. Photocatalyst Treatmentonto PET Fabrics

The standard white PET fabric was coated with the synthesized TiOF_2_ photocatalysts using pad-dry-cure method. As reported in the previous research, 3 wt.% of photocatalyst solution was the optimized concentration with which to coat the fabric [35]. Therefore, 3 wt.% of anatase TiO_2_ or TiOF_2_ in a water solution was prepared with 25 g of IPA and 1 g of PET fabric (9 cm × 9 cm) was dipped into the solution for 30 min. Then, the dipped fabric was padded to give a wet pick-up of 100%, dried at 90 °C for 30 min, and cured at 170 °C for 3 min. Finally, it was washed with IPA for 2 h and dried at 90 °C for 12 h. The prepared fabric samples were labeled based on the photocatalyst used as shown in Table 1.

### 2.4. Hydrophobization of the Photocatalyst Treated PET Fabrics

1 wt.% of hexadecyltrimethoxysilane (HDS) in IPA/water (9/1 = *w*/*w*) solution was prepared and stirred for 8 h at room temperature. The photocatalyst treated PET fabric was dipped into the prepared solution for 30 min. Then, the dipped fabric was padded to give a wet pick-up of 100% and dried at 90 °C for 30 min and cured at 170 °C for 3 min. Finally, it was washed with IPA for 2 h and dried at 90 °C for 12 h. The samples were labeled as Table 1.

### 2.5. Characterization of the Synthesized TiOF_2_ and Treated PET Fabrics

The crystallinity and structural properties of the synthesized TiOF_2_ were characterized using an X-ray diffractometer (XRD, Rikagu, D/Max-2500, Austin, TX, USA) equipped with Cu-Kα radiation at 40 kV and 200 mA. Quantitative analyses of TiOF_2_ and anatase TiO_2_ were carried out using Spurr and Myers relationship (A_(wt.%)_ = 1/(1 + 1.265 × (I_O_/I_F_)) × 100, where I_O_ is the integrated intensity of anatase TiO_2_ (101) at 25.3° and I_F_ is the integrated intensity of TiOF_2_ (100) at 23.4°) to calculate the phase compositions of the synthesized photocatalyst [37,38]. The average crystal sizes were also calculated using the Scherrer equation (L = 0.90λ/(β − βi) cos θ, where β is the observed FWHM (fulll width at half maximum) and is the calculated instrumental broadening) [37,38].

The morphologies of the TiOF_2_ and the treated PET fabrics were observed using a field-emission scanning microscope (FE-SEM, Hitachi SU8220, Tokyo, Japan) with magnifications of 2000 and 10,000.

The K/S values of the photocatalyst treated PET samples were measured from 460 to 760 nm using a spectrophotometer (CM-3600d, Konica Minolta, Tokyo, Japan) to confirm the visible light absorption of the synthesized photocatalysts.

### 2.6. Evaluation of Photocatalytic Self-Cleaning Properties of Treated PET Fabrics

Photocatalytic degradation of MB on the treated PET fabrics was performed to evaluate the photocatalytic self-cleaning properties of the fabrics. The fabric samples (9 cm × 9 cm) were first immersed in acetone, because the treated samples were too hydrophobic and an aqueous solution of MB (25 μM) could not wet the fabric. The acetone-immersed samples were dipped into 30 mL of the aqueous MB solution and shaken at 180 rpm for 8 h. Then, they were dried in the oven at 60 °C for 8 h. The prepared samples were exposed to UV or sunlight. In the UV degradation tests, a UV lamp (8W, 365 nm, Korea Ace Scientific, Seoul, Korea) was used and the prepared samples were placed 8 cm under the lamp for 0, 1, 4, 8, and 12 h. In the sunlight degradation test, since sunlight is hard to reproduce, all of the prepared samples in this study were tested at the same time. Three batches of samples prepared under the same conditions were assessed to confirm the reproducibility of the data. All of the samples were exposed to sunlight only from 10 a.m. to 4 p.m. on a sunny day and the exposure times were 0.5, 1, 4, 8, and 12 h. The K/S values of the tested samples were measured from 400 to 760 nm using a spectrophotometer (CM-3600d, Konica Minolta, Tokyo, Japan). The photodegradation properties of the tested samples were evaluated based on the Kubelka–Munk theory using the following calculation:

(1) $$Decomposition\;\left(\%\right)=\frac{{\left(\frac{K}{S}\right)}_{s}-{\left(\frac{K}{S}\right)}_{w}}{{\left(\frac{K}{S}\right)}_{s}-{\left(\frac{K}{S}\right)}_{0}}\times 100$$

(K/S)_0_ = K/S of non-stained fabric, (K/S)_S_ = K/S of MB-stained fabric, (K/S)_W_ = K/S of MB-stained fabric after light irradiation

### 2.7. Evaluation of Superhydrophobic Self-Cleaning Properties of Treated PET Fabrics

The superhydrophobic self-cleaning properties of the prepared samples were evaluated by measuring static and dynamic contact angles. The samples were pre-dried at 90 °C for 2 h. The static contact angles of the samples were measured by dropping 10 μL of water using a contact angle instrument (KRUSS DA100, Hamburg, German). The dynamic contact angles of the samples were measured using the laboratory-constructed goniometer to evaluate roll-off angles when 15 μL of water was dropped onto the fabric surface. Each measurement was repeated with three different samples.

### 2.8. Test of Practical Self-Cleaning Properties of Treated PET Fabrics

In the real textile industry, the textile finishing process follows the dying of fabrics. Therefore, the photocatalyst treatment and hydrophobization of dyed PET fabric were carried out as described in Section 2.3 and Section 2.4. The standard white PET fabric was dyed with C.I. disperse yellow 54 (LG Chemicals, Seoul, Korea) using 1% o.w.f. water solution with a liquor ratio of 1:40. Tomato ketchup was dropped on the dyed and treated fabric and left for 30 min. Then, the ketchup was wiped off the fabric surface and the contaminated fabric was exposed to sunlight for 0, 1, 2, and 4 h.

## 3. Results and Discussions

### 3.1. Characterization of the Prepared TiOF_2_

**Figure 1:**
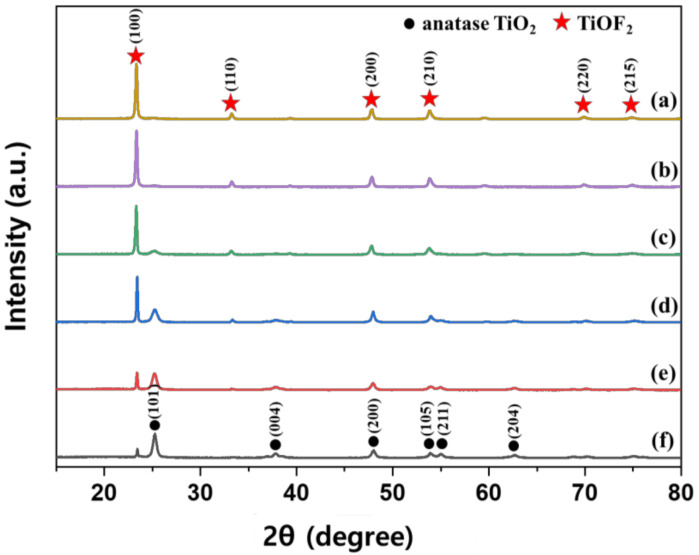
XRD patterns of the prepared TiOF_2_ at various reaction temperatures; (**a**) 300 °C, (**b**) 350 °C, (**c**) 400 °C, (**d**) 450 °C, (**e**) 500 °C and (**f**) 550 °C.

Figure 1 shows XRD patterns of the TiOF_2_ prepared at various reaction temperatures. Diffraction peaks corresponding to TiOF_2_ were observed at 23.4°, 33.3°, 39.5°, 48°, 55.1°, 60°, 69.9°, and 75.2° (JCPDS No. 01-0490); Figure 1a shows the data for 300 °C. As the reaction temperature increased, the diffreaction peaks decreased and were hard to observe, except for the peak at 23.4°. However, the diffraction peaks corresponding to anatase TiO_2_ at 25.3°, 37.8°, 47.8°, 53.9°, 55.0°, and 63.1° (JCPDS No. 21-1272) increased, as the reaction temperature increased. Phase composition of the prepared TiOF_2_ was shown in Table 2. When the reaction temperatures were 300, 350, and 400 °C, TiOF_2_ was dominant, whereas anatase TiO_2_ was dominant when the reaction temperature was 500 and 550 °C. When the reaction temperature was 450 °C, a mixture of TiOF_2_ and anatase TiO_2_ with a similar ratio was produced. Table 2 also shows the crystal sizes of the prepared photocatalysts calculated from the XRD results. The crystal sizes of anatase TiO_2_ gradually increased as the reaction temperature increased, whereas those of TiOF_2_ increased significantly up to 50%.

**Figure 2:**
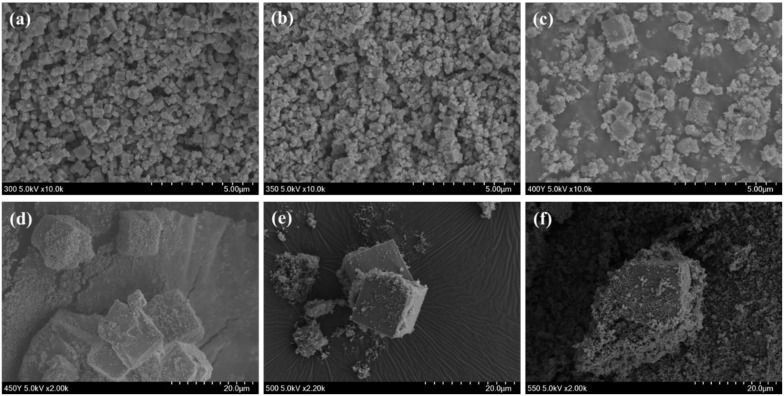
SEM images of the prepared TiOF_2_ at various reaction temperatures; (**a**) 300 °C, (**b**) 350 °C, (**c**) 400 °C, (**d**) 450 °C, (**e**) 500 °C and (**f**) 550 °C.

Figure 2 shows the SEM images of the prepared TiOF_2_ at various reaction temperatures; Cube-shaped particles were observed in all samples, suggesting the formation of TiOF_2_ [36]. The average particle sizes of the TiOF_2_ and TiO_2_ are also shown in Table 2. As shown in Figure 2 and Table 2, the particle size of TiOF_2_ itself dramatically increased from 0.40~0.47 to 9.74~14.20 µm, as the reaction temperature increased. However, when the reaction temperature was 300 or 350 °C, size of cube-shaped particles was less than 1 µm. The particle size of anatase TiO_2_ gradually increased from 81.5 to 107.5 nm, as the reaction temperature increased.

The XRD and SEM results show that the photocatalysts prepared at lower reaction temperatures contained more TiOF_2_ and were of smaller size than those prepared at higher reaction temperatures. This suggests that the photocatalytic degradation of theTiOF_2_ prepared at lower reaction temperatures should be superior. However, the color of the photocatalysts was dark black like carbon black, which hindered the observation of MB photodegradation on the fabric surface. Moreover, if the textile finishing agent had a deep color, it would change the color of the dyed fabric such that the fabric could not be practically used. Therefore, only the TiOF_2_ materials prepared at 450, 500, and 550 °C were further used to manufacture the self-cleaning fabrics.

### 3.2. Self-Cleaning Properties of Prepared Photocatalyst-Treated PET Fabrics

**Figure 3:**
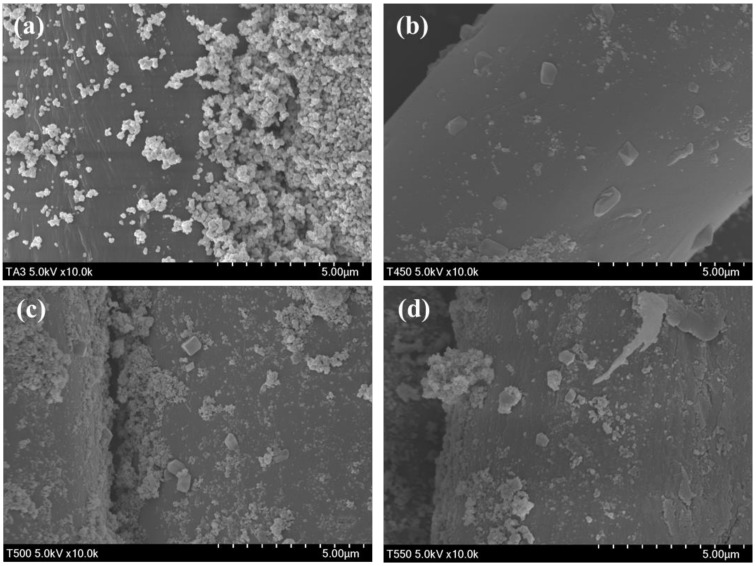
Morphologies of the prepared photocatalyst-treated PET fabrics; (**a**) aT, (**b**) 450TF, (**c**) 500 TF, and (**d**) 550 TF.

Figure 3 presents the morphologies of the prepared photocatalyst-treated PET fabrics. Sphere-shaped particles were observed in the SEM image of aT, whereas sphere-shaped particles and cube-like particles were observed in the SEM images of 450TF, 500TF, and 550TF. The size of the TiOF_2_ observed in 450TF, 500TF, and 550TF was much smaller (594.1~683.5 nm) than that of the TiOF_2_ powders (9.74~14.2 µm). This suggests that the size of TiOF_2_ decreased during the treatment to the PET.

**Figure 4:**
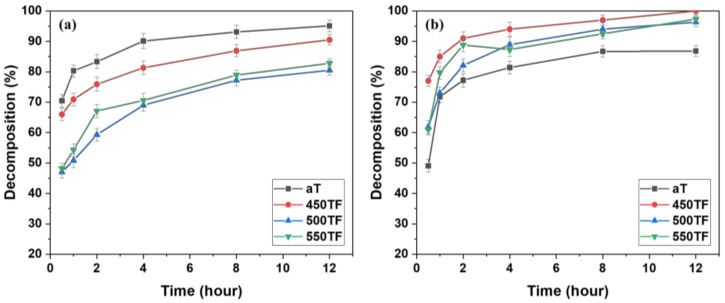
Photocatalytic self-cleaning properties of the photocatalyst-treated PET fabrics under (**a**) UV and (**b**) sunlight.

**Figure 5:**
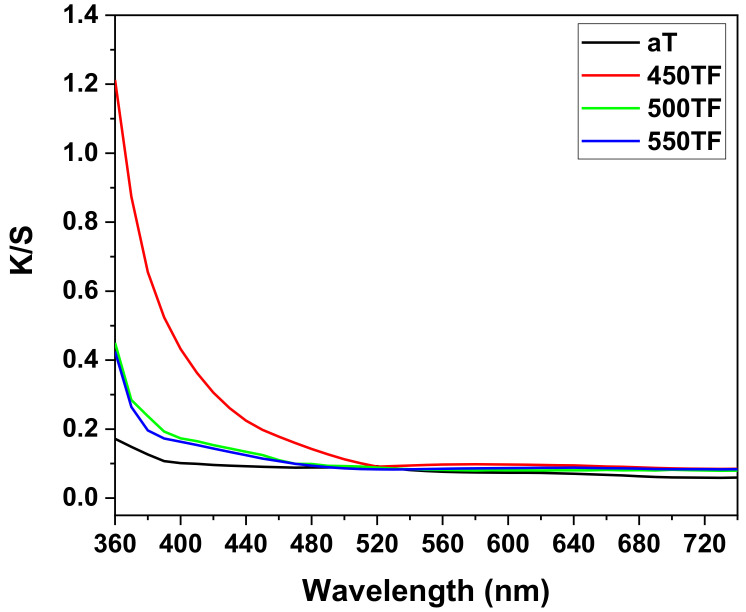
K/S values of fabrics aT, 450TF, 500TF, and 550TF.

Figure 4 shows the photocatalytic degradation of MB under UV and sunlight for the PET fabrics aT, 450TF, 500TF, and 550TF. With UV irradiation, aT exhibited the highest MB decomposition by photocatalytic degradation even after 30 min, and 95.1% of the MB stained on this fabric was decomposed after 12 h. However, with sunlight irradiation, aT exhibited the lowest level of MB decomposition with 86.8% decomposition, whereas 450TF, 500TF, and 550TF exhibited 100, 96.3, and 97.3% decomposition, respectively, after 12 h. Therefore, the photocatalytic self-cleaning properties of those samples were superior to the commercial anatase TiO_2_ treated samples. This is because anatase TiO_2_ only utilizes UV light, whereas TiOF_2_ utilizes both UV and visible light for the photodegradation of MB [39,40,41]. As already mentioned, UV makes up only a small portion of sunlight. To confirm visible light absorption of the prepared samples, the K/S values of aT, 450TF, 500TF, and 550TF from 360 to 780 nm were measured using a spectrophotometer mentioned in Section 2.6. Figure 5 shows that 450TF yielded significantly higher K/S values from 360 to 780 nm than the other fabrics with aT yielding the lowest K/S values. When the color was whiter, the K/S values from 360 to 780 nm were close to 0, whereas these values are higher, when the color was blacker. In other words, higher K/S values from 360 to 780 nm mean greater absorption of visible light. Therefore, 450TF showed the highest level of photodegradation owing to visible light absorption and sensitization.

**Figure 6:**
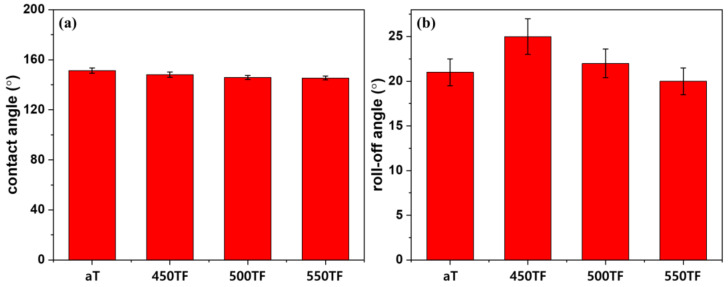
Superhydrophobic self-cleaning properties of the photocatalyst-treated PET fabrics; (**a**) contact angle and (**b**) roll-off angle.

Figure 6 depicts static and dynamic contact angles of the photocatalyst treated PET fabrics, which were used to evaluate superhydrophobic self-cleaning properties. Static contact angles were evaluated by measuring the contact angles of water droplet on the fabrics, whereas dynamic contact angles were evaluated by measuring roll-off angles of water droplet on the fabrics. Roll-off angles are especially important to evaluate practical self-cleaning properties, because the superhydrophobic self-cleaning of fabrics was based on the washing out of dirt by rolling water or liquid droplets. As shown in Figure 6a, water contact angles of the prepared fabrics were 145~151°, indicating superhydrophobicity was introduced by the surface roughening effect of the nanoparticles. The roll-off angles of the prepared fabrics were 20~25°. These values are greater than 10°, which is the maximum value considered to indicate superhydrophobic surface. Therefore, even if the photocatalyst-treated fabrics could be deemed superhydrophobic based on water contact angles, they were not sufficiently superhydrophobic to exhibit actual washing-off self-cleaning properties.

### 3.3. Self-Cleaning Properties of the Prepared Photocatalyst/HDS-Treated PET Fabrics

**Figure 7:**
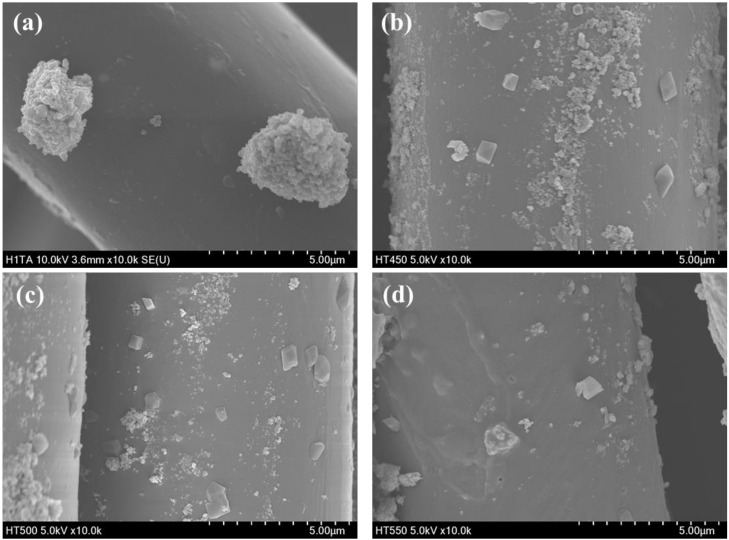
TMorphologies of the prepared photocatalyst/HDS-treated PET fabrics; (**a**) aT-H; (**b**) 450TF-H, (**c**) 500TF-H, and (**d**) 550TF-H.

Figure 7 shows the morphologies of the prepared photocatalyst/HDS-treated PET fabrics. Aggregated particle shapes were observed in the SEM image of aT-H, whereas more sphere-shaped particles were observed in the SEM images of 450TF-H, 500TF-H, and 550TF-H. The anatase TiO_2_ had surface -OH groups, which could function as reaction sites with HDS, whereas TiOF_2_ had no actual reaction sites. Therefore, HDS favorably attached to the surface of anatase TiO_2_, forming aggregated particle-like shapes on aT-H, whereas HDS favored attachment to the surface of PET fabric, forming some sphere or coated film shapes on 450TF-H, 500TF-H, and 550TF-H.

**Figure 8:**
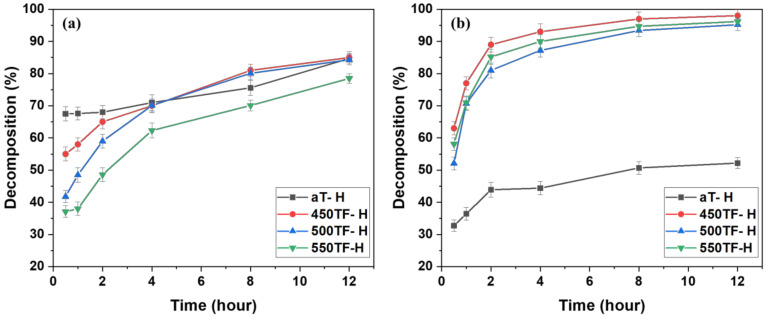
Photocatalytic self-cleaning properties of the photocatalyst/HDS-treated PET fabrics; under (**a**) UV and (**b**) sunlight.

Figure 8 depicts the photocatalytic degradation of MB under UV or sunlight for fabrics aT-H, 450TF-H, 500TF-H, and 550TF-H. With UV irradiation, aT-H exhibited the highest level of MB decomposition by photocatalytic degradation after only 30 min; however 450TF-H and 500TF-H showed the similar decomposition to that of aT-H after 12 h. With sunlight irradiation, aT exhibited the lowest level of MB decomposition with only 52.2% decomposition, whereas 450TF, 500TF, and 550TF exhibited 98, 95.2, and 96.2% decomposition, respectively, after 12 h. Therefore, the photocatalytic self-cleaning properties of those samples were superior to the commercial anatase TiO_2_/HDS-treated samples, as shown in Section 3.2. This is quite impressive, because we expected that HDS would cover the surface of the photocatalyst on the fabric surface and significantly hinder photocatalytic degradation. However, the surface of the photocatalyst were not perfectly covered, allowing it to maintain its function.

**Figure 9:**
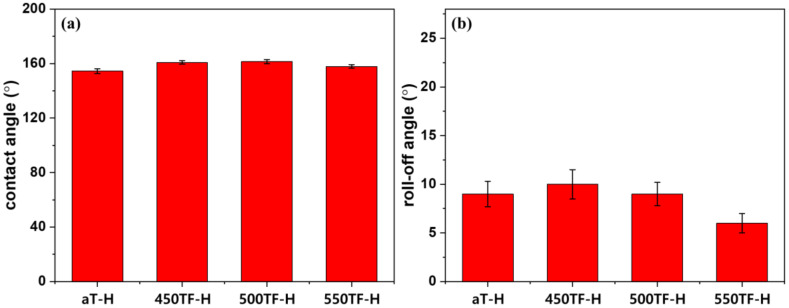
Superhydrophobic self-cleaning properties of the photocatalyst/HDS-treated PET fabrics; (**a**) contact angle and (**b**) roll-off angle.

Figure 9 depicts the static and dynamic contact angles of the photocatalyst/HDS-treated PET fabrics, which were used to evaluate superhydrophobic self-cleaning properties. As shown in Figure 9a, water contact angles of the prepared fabrics were 154~158°, indicating that the superhydrophobicity of the fabrics increased after HDS treatment via the surface roughening effect of the nanoparticles and the surface energy lowering effect of HDS. The roll-off angles of the prepared fabrics were 6~10° as shown in Figure 9b. Therefore, these fabrics showed excellent superhydrophobicity, i.e., sufficient to exhibit actual washing-off self-cleaning properties, due to the synergistic effect of the photocatalyst and HDS in optimizing the surface roughness.

These results suggest that self-cleaning PET fabrics with superhydrophobicity and photocatalytic degradation could be prepared using TiOF_2_/HDS treatment.

### 3.4. Practical Self-Cleaning Properties of the Photocatalyst/HDS-Treated Fabric

**Figure 10:**
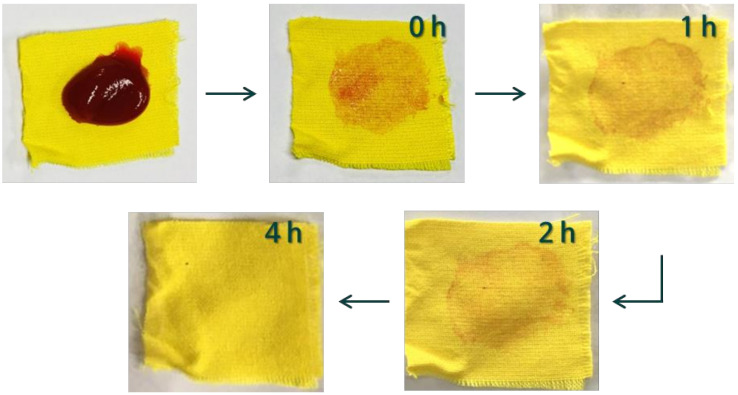
Ketchup stain self-cleaning of the 450TF-H PET fabric.

As mentioned earlier, the self-cleaning treatment is usually applied to dyed fabric. When dyed fabric is used, it is important to retain usable color even after the treatment. Therefore, for the first time, we assessed the self-cleaning properties of the dyed PET fabric. Figure 10 shows the self-cleaning ability of the photocatalyst/ HDS-treated PET fabric (450TF-H) toward a ketchup stain. As shown in Figure 10, there was no significant color issue after the treatment. Color issues can potentially occur when using a visible light sensitizing photocatalyst because the photocatalyst itself could have color owing to visible light absorption. Before sunlight exposure of 450TF-H, it was observed that the ketchup stain was hardly formed on the surface due to its superhydrophobicity. We also tried coffee and wine applications and no noticeable stain was observed. The ketchup was oilier than the coffee and wine, so a noticeable stain was obtained. After 4 h of exposure to sunlight, no noticeable ketchup stain was observed on the surface of the fabric. These results suggest that practical self-cleaning PET fabrics with superhydrophobicity and photocatalytic degradation could be prepared using the TiOF_2_/HDS treatment.

## 4. Conclusions

In this study, self-cleaning PET fabrics were prepared using TiOF_2_/HDS treatment. The prepared PET fabrics had superior photocatalytic self-cleaning properties to anatase TiO_2_/HDS-treated PET fabrics under UV and sunlight with 98% decomposition of MB. TiOF_2_/HDS-treated PET fabrics also had superior superhydrophobic self-cleaning properties to anatase TiO_2_/HDS-treated PET fabrics with a 161° water contact angle and 6° roll-off angle. After the self-cleaning tests of undyed TiOF_2_/HDS-treated PET fabrics, we prepared dyed TiOF_2_/HDS-treated PET fabrics to test the practical aspect of the treatment method. These PET fabrics were hardly stained with tomato ketchup; even when they were stained, they self-cleaned after 4 h.

These results suggest that practical self-cleaning PET fabrics with superhydrophobicity and photocatalytic degradation could be prepared using TiOF_2_/HDS treatment. However, the synthesis of TiOF_2_ was not practical due to the use of fluorine gas and high temperature. Moreover, the durability of the treatment was not investigated and more study is required prior to the application of these findings to the textile industry.

## Figures and Tables

**Table 1:** Sample labels used in this study based on preparation conditions.

Sample	Treatment Method
aT	PET treated with the commercial anatase TiO_2_
450TF	PET treated with TiOF_2_ synthesized at 450 °C
500TF	PET treated with TiOF_2_ synthesized at 50 °C
550TF	PET treated with TiOF_2_ synthesized at 550 °C
aT-H	Hexadecyltrimethoxysilane treated aT
450TF-H	Hexadecyltrimethoxysilane treated 450TF
500TF-H	Hexadecyltrimethoxysilane treated 500TF
550TF-H	Hexadecyltrimethoxysilane treated 550TF

**Table 2:** Phase composition and crystal size analysis from XRD data in Figure 1 and average particle size from SEM data in Figure 2.

Temperature of Synthesis (°C)	Phase Composition ^a^		Crystals Size From XRD ^b^		Particle Size From SEM	
	TiOF_2_ (wt.%)	Anatase TiO_2_ (wt.%)	TiOF_2_ (nm)	Anatase TiO_2_ (nm)	TiOF_2_ (μm)	Anatase TiO_2_ (nm)
300	94.2	5.8	44	11	0.40 ± 0.09	81.5 ± 10.7
350	93.7	6.3	46	13	0.46 ± 56.7	88.0 ± 5.6
400	80.0	20.0	48	17	0.47 ± 139.5	91.0 ± 7.3
450	43.0	57.0	64	17	9.74 ± 1.70	105.6 ± 5.4
500	19.7	80.3	62	18	10.07 ± 1.54	107.5 ± 8.0
550	7.8	92.2	66	20	14.20 ± 1.41	105.1 ± 3.6

^a^ Calculated by Spurr and Myeres relation. ^b^ Calculated by Scherrer’s equation.
